# Complete genome assembly and functional characterization of *Brucella melitensis* strain IMHB1 from a clinical isolate in Inner Mongolia, China

**DOI:** 10.3389/fcimb.2025.1653521

**Published:** 2025-12-09

**Authors:** Xu Zheng, Chen Liang, Liping Shao, Chenfang Liu, Ruiyuan Yao, Li Peng, Yanyu Liang, Xiuwen Liang, Shiyong Liu

**Affiliations:** 1 Brucellosis Clinical Laboratory, the Hulunbuir People’s Hospital, Hulunbuir, China; 2 Hulunbuir Clinical Medical College, Inner Mongolia Minzu University, Tongliao, China; 3 Basic Medicine College, Inner Mongolia Medical University, Hohhot, China; 4 Brucellosis Department, Hulunbuir Zhongmeng Hospital, Hulunbuir, China

**Keywords:** whole-genome sequencing, *Brucella melitensis*, genomic features, function analysis, virulence factors

## Abstract

Brucellosis is a globally prevalent zoonotic disease caused by *Brucella* species, posing a significant threat to both public health and the livestock industry. Despite ongoing research efforts, the mechanisms underlying *Brucella* pathogenesis remain poorly understood, particularly for strains isolated from specific geographical regions. A *Brucella melitensis* biotype III strain, IMHB1, was isolated from the blood culture of a patient in Hulunbuir, Inner Mongolia, China, who had experienced multiple relapses of brucellosis. Using Oxford Nanopore long-read sequencing, a complete 3.32 Mbp genome was assembled comprising two circular chromosomes with a GC content of 57.22% and 3,152 predicted coding sequences. Phylogenetic analysis revealed that IMHB1 was closely related to the cgST-588 type. Comprehensive genomic characterization identified mobile genetic elements, horizontally transferred regions, and prophage insertions. Functional annotation detected 10 genomic islands, 45 carbohydrate-active enzymes, 3 biosynthetic gene clusters, 4 antibiotic resistance genes, 20 eggNOG categories, and 252 KEGG pathways. Moreover, 66 predicted virulence factors and 18 experimentally verified proteins associated with pathogen-host interactions were identified, suggesting their potential roles in virulence and host adaptation. Based on extensive bioinformatics analysis, this study provides novel insights into the genomic characteristics and potential pathogenic mechanisms of *Brucella melitensis* strain IMHB1, enriching existing genomic resources and contributing to future research on brucellosis pathogenesis and therapeutic strategies.

## 1 Introduction

Brucellosis is a globally contagious zoonosis caused by *Brucella* spp., imposing significant human and economic burdens. Over 500,000 new human cases are reported annually worldwide (Corbel, 2016), and a meta-analysis of surveillance data estimated the annual number of cases could reach up to 2.1 million, highlighting ongoing underreporting (Laine et al., 2023). Recent data indicate that the prevalence of brucellosis has expanded from 53 to at least 97 countries, with Kenya exhibiting the highest incidence, reaching 293.1 cases per 100,000 in 2019 (Liu et al., 2024). The disease causes significant losses to agriculture, animal husbandry, public health, and the social economy. *Brucella*, a facultative intracellular gram-negative coccobacillus, is the causative agent of brucellosis. Twelve *Brucella* spp. have been described, including *Brucella melitensis*, *B. abortus*, *B. suis*, *B. ovis*, *B. canis*, *B. neotomae*, *B. ceti*, *B. pinnipedialis*, *B. microti*, *B. inopinata*, and *B. papionis* (Whatmore et al., 2014; Scholz et al., 2016). Among these, *B. melitensis* is the most common human-infecting species and exhibits high virulence (Głowacka et al., 2018). Unlike many pathogens, *Brucella* lacks exotoxins and plasmid-encoded toxins but employs stealth strategies to persist intracellularly (Barquero-Calvo et al., 2007; Conde-Álvarez et al., 2012). Its key virulence characteristics include prolonged survival within host cells and evasion of the host immune system, ultimately leading to chronic infection.

*Brucella* is primarily transmitted to humans through the consumption of unpasteurized dairy products or direct contact with infected animals (Corbel, 2016). It is highly infectious, with an aerosol dose as low as 10–100 organisms (Bossi et al., 2004). Upon infection with *Brucella*, humans exhibit nonspecific symptoms such as fever, headache, arthralgia, myalgia, fatigue, and sweating. Despite extensive research, the mechanisms by which *Brucella* induces pathological changes across organs remain poorly understood. Additionally, genomic data for *Brucella* isolates from Inner Mongolia are limited, hindering region-specific control strategies. A comprehensive understanding of *Brucella*’s biological characteristics is therefore essential to elucidate its pathogenic processes and mechanisms.

Advances in sequencing technologies, such as Illumina and Oxford Nanopore, coupled with the development of refined and widely adopted databases, have greatly accelerated genomic studies of bacterial pathogens. For instance, the virulence factor database (VFDB) is used for virulence factor identification, the comprehensive antibiotic resistance database (CARD) for antibiotic resistance gene screening, and Proksee for genome assembly and visualization (Liu et al., 2022; Alcock et al., 2023; Grant et al., 2023). However, at the time of writing, only 387 *Brucella* genomes at the chromosomal and complete assembly levels were available in the national center for biotechnology information (NCBI) Genome database, compared with 7,660 for *Escherichia coli* and 2,904 for *Staphylococcus aureus*, indicating that research on *Brucella* has received limited attention.

The first *B. melitensis* 16M genome, published in 2002, provided foundational insights into the structural and functional characteristics of this species (DelVecchio et al., 2002). Advances in long-read sequencing technologies now enable high-quality *de novo* assembly of *Brucella* genomes, revealing >97% sequence similarity among strains (DelVecchio et al., 2002). Nevertheless, individual strains exhibit distinct virulence, host preferences, and zoonotic potential (Suárez-Esquivel et al., 2017; Sheppard et al., 2018), and their clustering patterns correspond to the geographical region of the preferred host (Moreno, 2014; Vergnaud et al., 2018; Suárez-Esquivel et al., 2020).

We present the complete genome sequence of the clinical isolate IMHB1 from Inner Mongolia and perform comparative analyses to identify its genomic features and virulence determinants. *B. melitensis* strain IMHB1 was isolated from the blood of a patient with brucellosis in Hulunbuir, Inner Mongolia, China, and identified by matrix-assisted laser desorption/ionization time of flight mass spectrometry (MALDI-TOF MS), AMOS PCR assay, and monospecific agglutination tests. The genome was sequenced and *de novo* assembled, followed by core-genome multilocus sequence typing (cgMLST) genotyping, structural characterization, and functional annotation. Biological pathways and pathogenic molecules were analyzed and predicted, providing a foundation for investigating the pathogenic mechanisms of *Brucella*.

## 2 Materials and methods

### 2.1 Clinical information

A 55-year-old male patient presented with low-grade fever, excessive sweating, and fatigue for >20 days and had experienced multiple episodes of brucellosis over the past 5 years. The patient was diagnosed with *Brucella* infection using the Rose Bengal plate agglutination test (RBPT), serum agglutination test (SAT), and blood culture according to the Diagnostic Criteria for Brucellosis issued by the Health Department of the People’s Republic of China (National Health Commission of the People’s Republic of China, 2019). The RBPT result was positive, and the SAT titer was 1:400++. The blood culture flagged positive at 2.98 days, and based on the growth curve characteristics, *Brucella* growth was strongly suspected. The patient received intravenous doxycycline and levofloxacin for 14 days, resulting in significant clinical improvement and a negative blood culture, and the patient was followed by oral administration of both drugs for an additional 6 weeks.

### 2.2 Ethics statement

The patient was informed about the purpose and procedures of this study and provided written informed consent. All experimental procedures were approved by the Ethics Committee of Hulunbuir People’s Hospital.

### 2.3 Genomic and microbiological techniques

**Figure 1:**
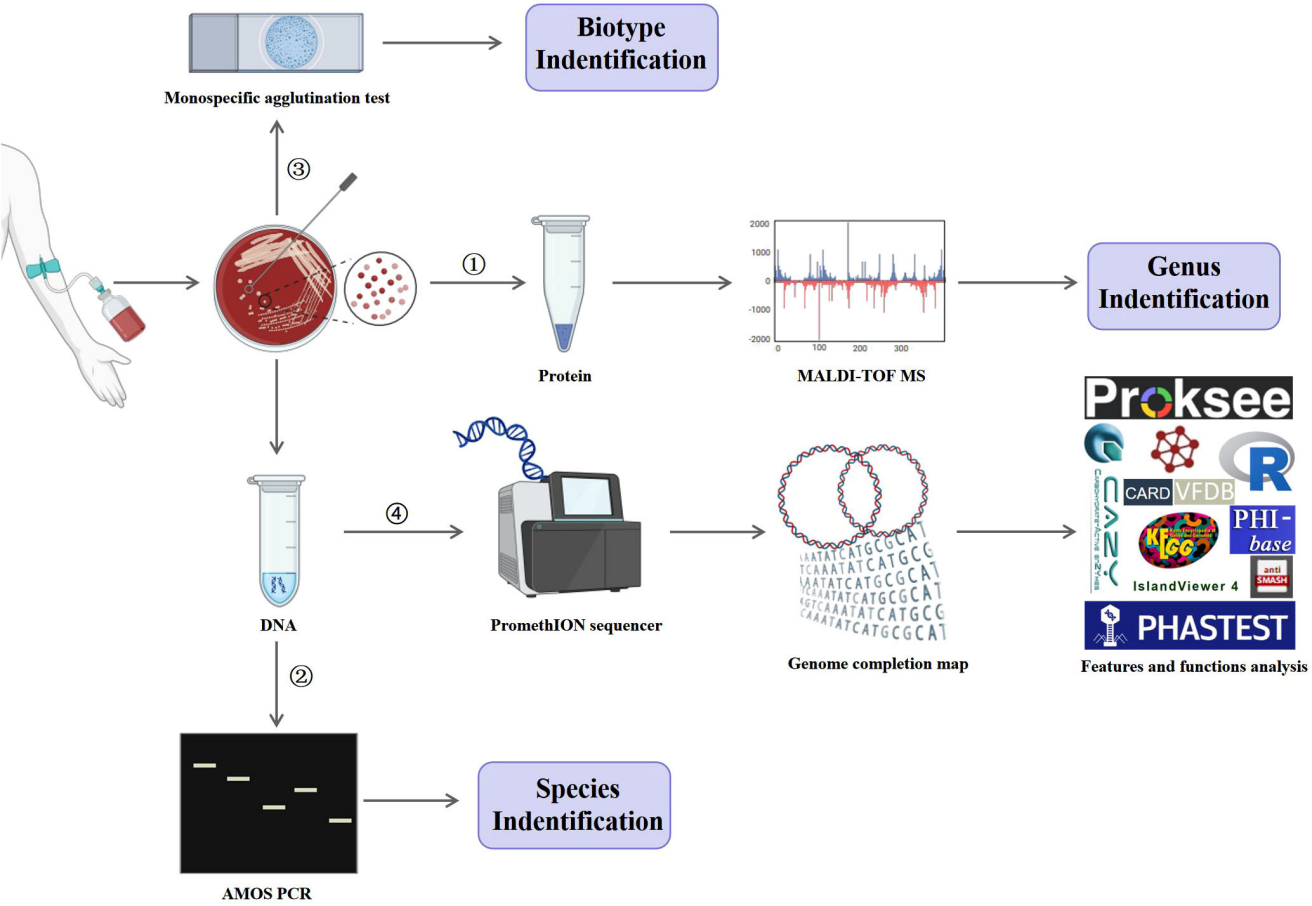
Experimental procedures. Strain IMHB1, isolated from the blood of a patient with brucellosis, was identified at the genus, species, and biotype levels by MALDI-TOF MS, AMOS PCR, and monospecific agglutination test, respectively. Genomic DNA of strain IMHB1 was then sequenced using the PromethION and Illumina NovaSeq platforms, followed by genome assembly and completion mapping. The genomic features and functional annotations of strain IMHB1 were analyzed using PubMLST, Proksee, R software, PHASTEST, IslandViever 4, CAZy, CARD, antiSMASH, eggNOG, KEGG, VFDB, and PHI-base databases.

Strain IMHB1, isolated from the patient’s blood, was identified at the genus, species, and biotype levels by MALDI-TOF MS, AMOS PCR, and monospecific agglutination tests, respectively. As illustrated in **Figure 1**, the DNA of strain IMHB1 was sequenced on PromethION sequencer and Illumina NovaSeq platforms, and a complete genome map was constructed. Genome features and functions were analyzed using Proksee (Grant et al., 2023), R software (R Core Team, 2022), and specialized functional tools and databases, including public databases for molecular typing and microbial genome diversity (PubMLST) (Jolley et al., 2018), phage search tool with enhanced sequence translation (PHASTEST) (Wishart et al., 2023), an integrated interface for computational identification and visualization of genomic islands (IslandViewer 4) (Bertelli et al., 2017), carbohydrate-active enzymes (CAZy) database (Lombard et al., 2024), CARD (Alcock et al., 2023), antibiotics and secondary metabolite analysis shell (antiSMASH) (Blin et al., 2023), evolutionary genealogy of genes: non-supervised orthologous groups (eggNOG) database (Huerta-Cepas et al., 2018), kyoto encyclopedia of genes and genomes (KEGG) database (Kanehisa and Goto, 2000), VFDB (Liu et al., 2022), and pathogen host interactions (PHI-base) database (Urban et al., 2022).

### 2.4 Bacterial isolation and identification

Strain IMHB1 was obtained from the blood of a patient with brucellosis at Hulunbuir People’s Hospital (Hulunbuir, Inner Mongolia, China). Blood (10 mL) was collected in a disposable culture bottle containing 30 mL of compounded medium with polymeric adsorbent beads (bioMérieux, Lyon, Rhone, France). The medium comprised of peptone, anticoagulants, vitamins, amino acids, carbon sources, and trace elements, and the bottles were filled with N_2_, O_2_, and CO_2_. The blood-containing bottles were incubated in a BACT/ALTERT 3D 240 automated culture instrument (bioMérieux, Lyon, Rhone, France) and monitored for microbial growth over 7 days by performing optical inspection every 10 minutes.

Growth was detected after 2.98 day by an automated culture instrument, after which the culture was transferred to Columbia blood agar plates (Autobio, Zhengzhou, Henan, China) and incubated at 37°C with 5% CO_2_ for 5 days. Protein extraction was performed to identify the bacterial genus. followed by MALDI-TOF MS (Zybio, Chongqing, China). DNA was extracted using a QIAGEN kit (Hilden, Germany) for species identification via AMOS PCR. Colonies were harvested for biotype determination using monospecific serum A and M agglutination tests (Tsingtao Sinova HK Biotechnology, Qingdao, Shandong, China).

### 2.5 AMOS PCR amplification

The extracted DNA was subjected to multiplex AMOS PCR (TransGen, Beijing, China). Primer sequences are listed in **Table 1**, and all primers were synthesized by Sangon Biotech Co., Ltd. (Shanghai, China). Primer P_IS711_ was used at a final concentration of 1 µM, and other primers at 0.2 µM. The PCR program was 95°C for 5 min, 30 cycles at 95°C for 1 min, 60°C for 1 min, and 72°C for 1 min, followed by 72°C for 10 min.

**Table 1:** Oligonucleotide primers used in the AMOS-PCR assay.

Primer name	Sequence (5´→3´)	Target	Amplicon size (bp)
P_A_	GACGAACGGAATTTTTCCAATCCC	*B. abortus*	498
P_M_	AAATCGCGTCCTTGCTGGTCTGA	*B. melitensis*	731
P_O_	CGGGTTCTGGCACCATCGTCG	*B. ovis*	976
P_S_	GCGCGGTTTTCTGAAGGTGGTTCAGG	*B. suis*	285
P_IS711_	TGCCGATCACTTAAGGGCTTCAT	–	–

### 2.6 Sequencing and genome assembly

The extracted DNA was quantified using a Qubit 3.0 fluorometer (ThermoFisher Scientific, Waltham, Massachusetts, U.S.A.) at 67 ng/μL. Purity was assessed by NanoDrop spectrophotometry (ThermoFisher Scientific, Waltham, Massachusetts, U.S.A.), with OD260/280 and OD260/230 ratios of 1.94 and 1.89, respectively. Fragment size analysis indicated that the predominant DNA fragments were greater than 20 kb.

DNA was purified by magnetic bead separation, damage-repaired, end-repaired, and ligated to barcode tags and sequencing adapters. The processed DNA was loaded onto an R9.4 sequenced chip for genomic sequencing on a PromethION sequencer using Oxford Nanopore long-read sequencing (Oxford Nanopore Technologies, Oxford, UK) and yielded an average depth of 591× relative to this genome. Simultaneously, short-read data were generated to create a high-quality draft assembly on an Illumina NovaSeq platform using Illumina short-read sequencing (Illumina, San Diego, California, U.S.A.), yielding an average depth of 448× coverage. Sequencing was performed by OE Biotech Co. Ltd. (Shanghai, China). Unicycler v0.4.9 was used for genome assembly, and Pilon v1.23 was employed to correct errors and generate high-accuracy assembled genomic data. Assembly quality was assessed with QUAST v5.0, achieving an N50 of 2,126,219 bp.

### 2.7 Genomic data collection

Fourteen *Brucella* genomes were retrieved from the NCBI Genome database on October 12, 2025, including two reference strains (*B. melitensis* 16M and *B. abortus* 554) and 12 strains isolated in China. The genomes of the isolated strains were selected based on the following criteria: *B. melitensis*, complete assemblies, with clearly documented host and geographical origin. Detailed information on each genome, including the GenBank assembly ID, species, host, and geographical location, is provided in **Supplementary Table S1**.

### 2.8 cgMLST and Phylogenetic reconstruction

cgMLST was performed for all 15 *Brucella* genomes using the schemes available on the PubMLST database (https://pubmlst.org/brucella/). The obtained allelic profiles were used to construct a pairwise Manhattan distance matrix representing the number of allelic mismatches between strains. A phylogenetic tree was subsequently constructed from this distance matrix using the Neighbor-Joining (NJ) algorithm in R v4.5.1 with the ape package v5.8-1. The robustness of the phylogenetic inference was evaluated with 1,000 bootstrap replicates. The final tree was visualized and annotated using the ggtree package v3.16.3.

### 2.9 Bioinformatics analysis

The integrated Bakta (Schwengers et al., 2021) v1.9 (httpsgithubcomoschwengersbakta), a command-line application for bacterial genome annotation, was applied to analyze the genomic structural characteristics using default parameters. Built-in tools within Bakta include Prodigal for predicting coding sequences (CDSs), tRNAscan-SE for transfer RNAs (tRNAs), Aragorn for transfer-messenger RNAs (tmRNAs), Infernal for ribosomal RNAs (rRNAs) and non-coding RNAs (ncRNAs), CRISPRCasFinder for clustered regularly interspaced short palindromic repeats (CRISPR), and NCBI BLAST+ v2.16 for identifying the origin of replication. Repeat elements were predicted using RepeatModeler v2.0.5 and RepeatMasker v4.1.5. The genomic circular map was visualized online using the Proksee system (https://proksee.ca/).

Potential horizontal gene transfer events were predicted using Alien Hunter (Vernikos and Parkhill, 2006) v1.7 with default parameters. Mobile genetic elements were annotated using MobileOG-db v1.1. Phage genomic sequences were predicted online using the PHASTEST database in deep mode (https://phastest.ca/). Genomic islands (GIs) were identified using IslandViewer 4 (https://www.pathogenomics.sfu.ca/islandviewer/) with default bacterial genome settings.

Carbohydrate-active enzymes (CAZymes) were predicted using HMMER against the database for carbohydrate-active enzyme notation (dbCAN) (https://bcb.unl.edu/dbCAN2/) and DIAMOND against the CAZy database (https://www.cazy.org/). Antimicrobial resistance genes were identified using RGI v6.0 against the CARD (https://card.mcmaster.ca/) under strict criteria. Biosynthetic gene clusters (BGCs) were predicted using antiSMASH v7.0 with default bacterial settings.

The complete assembly of *B. melitensis* strain IMHB1 was functionally annotated using eggNOG-mapper v2.0 against the eggNOG database (https://eggnogdb.org/) with default parameters. Annotation was performed in HMMER mode to assign COG and KEGG pathway functional categories, and results were visualized using the ggplot2 package v3.5.1 in R v4.5.1. Virulence factors were annotated using NCBI BLAST+ v2.16 against the VFDB core database (http://www.mgc.ac.cn/VFs/) with default parameters. Interactions between the pathogen and host were analyzed using NCBI BLAST+ v2.16 against the PHI database (http://www.phi-base.org/) with default parameters.

## 3 Results

### 3.1 Isolation and identification of strain IMHB1

**Figure 2:**
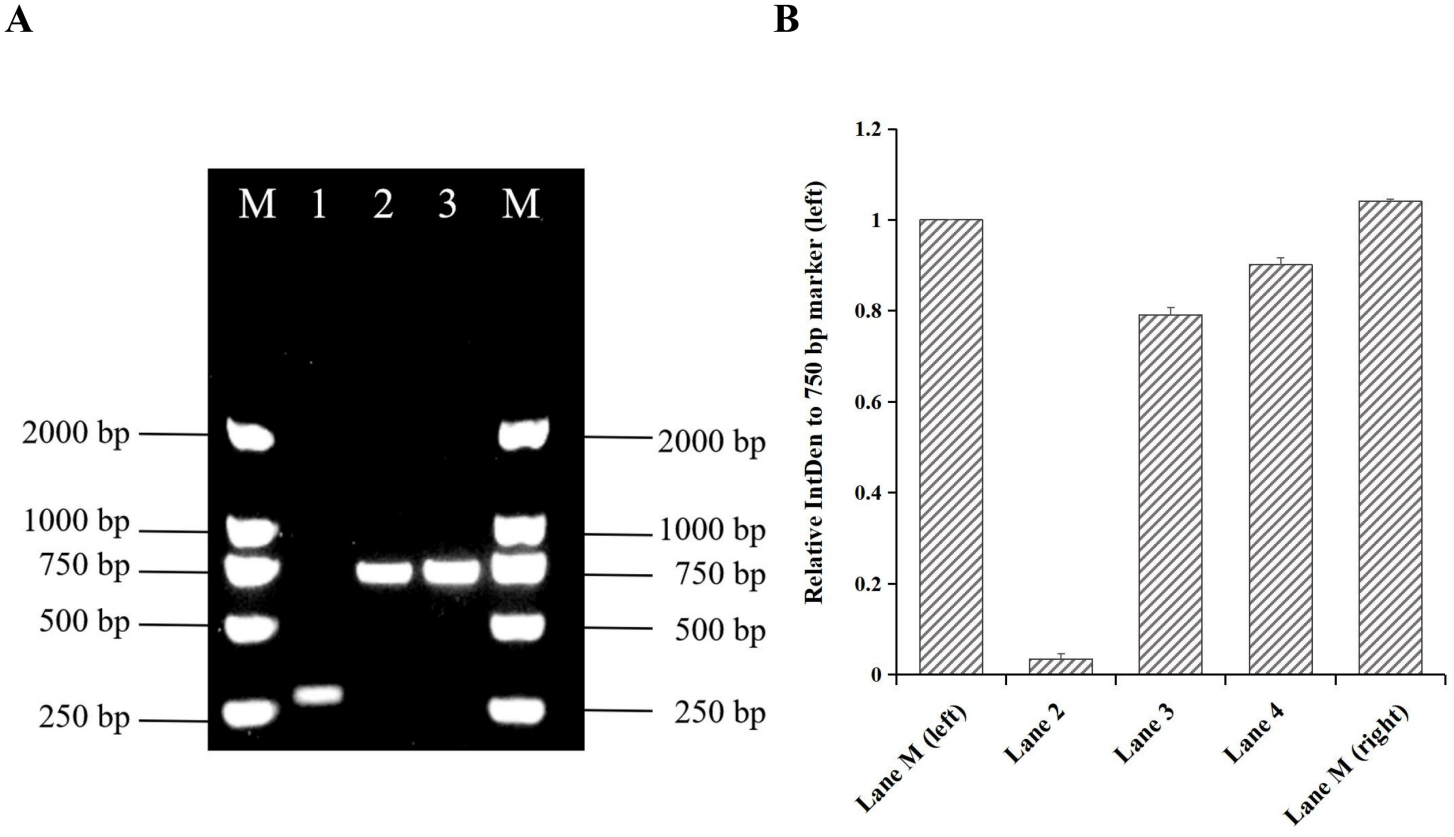
Identification of strain IMHB1 by AMOS PCR. **(A)** Amplified fragment of strain IMHB1 obtained by AMOS PCR. Lane M: *Trans*2K DNA marker; Lane 1: *Brucella suis* strain S2; Lane 2: *Brucella* strain IMHB1; Lane 3: *Brucella melitensis* strain M5. **(B)** Relative integrated density (IntDen) of the 750 bp band (left) in each lane. The integrated density of the DNA band was quantified three times using ImageJ2, and data were expressed as the mean ± standard deviation (SD).

A blood sample (internal ID: 125013166875) collected on January 31, 2025, was cultured in an automated culture instrument which detected a positive signal for microbial growth after 2.98 days. The culture was then transferred to a Columbia blood agar plate and incubated at 37°C and 5% CO_2_ for 5 days. The isolate was identified as a member of the *Brucella* genus by MALDI-TOF MS, with a log(score) ≥2.0 indicating genus-level identification. Based on the AMOS PCR amplicon size shown in **Table 1**, the amplified fragment (750 bp) corresponded to that of *B. melitensis* strain M5, suggesting that strain IMHB1 belongs to *B. melitensis* (**Figure 2A**). The relative integrated density of the 750 bp band in each lane was quantified using ImageJ2 to confirm specificity (**Figure 2B**). Agglutination tests with *B.* monospecific sera A and M were positive, confirming that the *B. melitensis* isolate belongs to biotype III. This strain was designated IMHB1, indicating its origin from Hulunbuir, Inner Mongolia.

### 3.2 cgMLST genotyping

**Figure 3:**
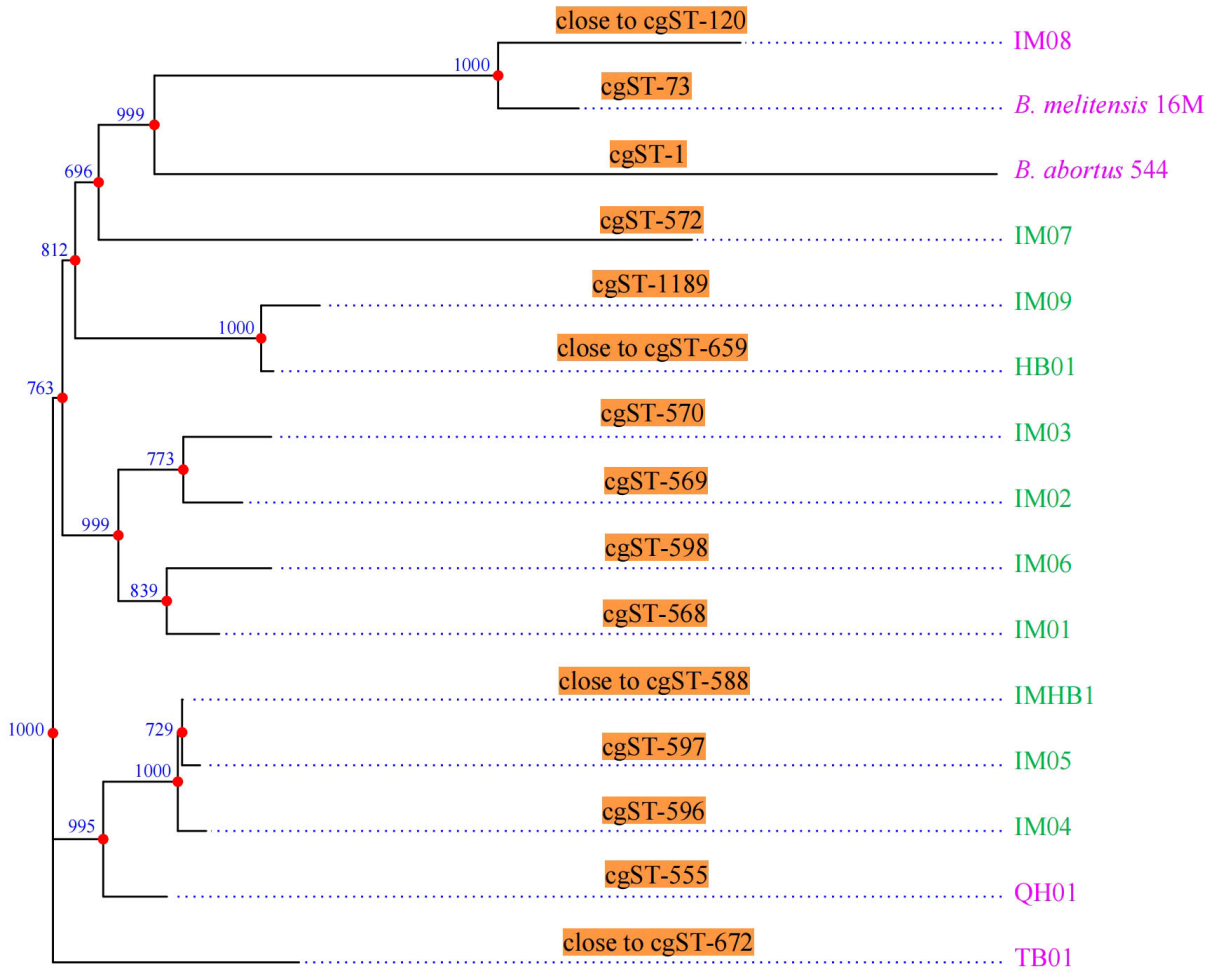
CgMLST genotyping and phylogenetic analysis. CgMLST analysis resolved the 15 strains into 15 distinct cgSTs. A phylogenetic tree constructed based on the cgMLST allelic profiles grouped these strains into nine primary clusters. Green represents strains isolated from humans, whereas pink represents strains isolated from animals. Phylogenetic inference robustness was evaluated using 1,000 bootstrap replicates, with bootstrap values shown at each branch point.

The comparative cgMLST analysis of 15 *Brucella* genomes from the PubMLST database, including strain IMHB1, revealed 15 distinct cgMLST sequence types (cgSTs), demonstrating substantial genetic diversity among the isolates. Strain IMHB1 showed no exact match in the database; its closest related profile was cgST-588, differing at five loci. The phylogenetic tree based on cgMLST allelic profiles grouped the 15 strains into nine primary clusters (**Figure 3**). Within this phylogeny, IMHB1 exhibited a close evolutionary relationship with two other human-derived *B. melitensis* strains from Inner Mongolia, whereas a sheep-derived strain from the same region was most closely related to the reference strain *B. melitensis* 16M.

### 3.3 Genomic structural characteristics of strain IMHB1

Genomic DNA from *B. melitensis* strain IMHB1 was sequenced using Oxford Nanopore long-read and Illumina short-read technologies. *De novo* assembly achieved 591× Nanopore and 448× Illumina coverage, with an N50 of 2.13 Mbp (Nanopore) and a Q30 of 98.31% (Illumina). BUSCO v5.3.2 analysis against the Rhizobiales odb10 dataset indicated 99.8% completeness. Sequencing and assembly statistics are summarized in **Supplementary Table S2**, demonstrating high accuracy of base recognition and genome assembly.

**Figure 4:**
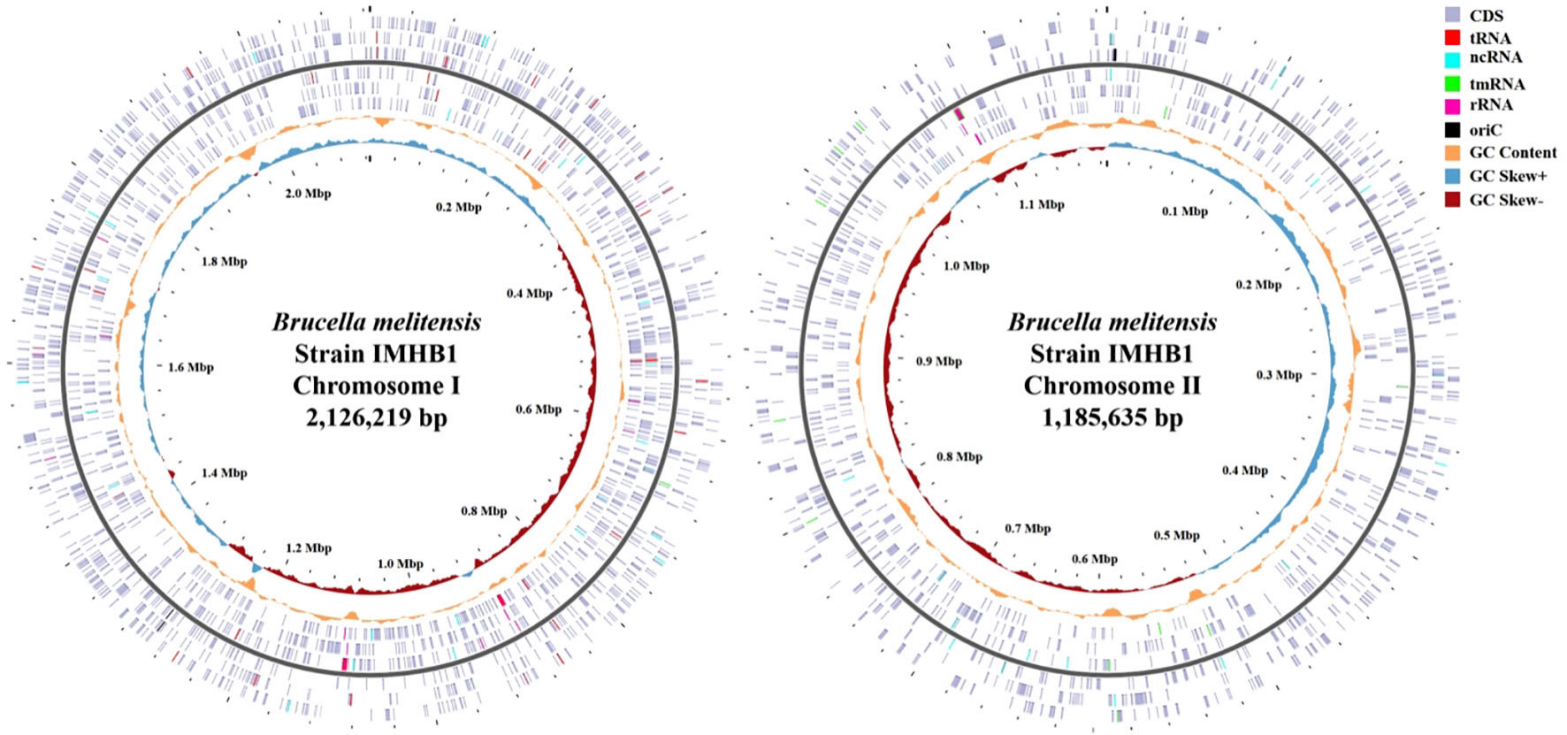
Completed genomic circle map of *Brucella melitensis* strain IMHB1. Left: Complete genomic circle map of chromosome I, comprising 2,038 CDSs, 41 tRNAs, one tmRNA, six rRNAs, 27 ncRNAs, and two oriCs. Right: Complete genomic circle map of chromosome II, comprising 1114 CDSs, 14 tRNAs, three rRNAs, 12 ncRNAs, and one oriC. The light purple block represents CDS; red, tRNA; bright blue, ncRNA; green, tmRNA; rose pink, rRNA; black, oriC; the orange-yellow curve, GC content; the light blue curve, GC skew (+); and dark red curve, GC skew (-).

As shown in **Tables 2** and **3**, the genome of *B. melitensis* strain IMHB1 consisted of two circular chromosomes measuring 2.13 Mbp and 1.19 Mbp, respectively. Chromosome I contained 2038 CDSs, 41 tRNAs, 1 tmRNA, 6 rRNAs, 27 ncRNAs, 80 repeat elements, and 2 oriCs, with a total coding length of 1.82 Mbp. Chromosome II comprised 1114 CDSs, 14 tRNAs, 3 rRNAs, 12 ncRNAs, 38 repeat elements, and 1 oriC (all open reading frames [ORFs] are shown in the genomic circle maps, **Figure 4**). Coding sequences accounted for 86% of chromosome I and 88% of chromosome II, with 959 and 940 genes per Mbp, respectively. These data reveal strong structural and functional consistency between strain IMHB1 and *B. melitensis* 16M.

**Table 2:** Statistical data of the genome and predicted genes of *Brucella melitensis* strains IMHB1 and 16M.

Category	IMHB1		16M	
	Chromosome I	Chromosome II	Chromosome I	Chromosome II
Genome size (bp)	2,126,219	1,185,635	2,117,144	1,177,787
Total gene length (bp)	1,823,751	1,042,608	1,814,604	1,032,489
Intergenetic region length (bp)	302,468	143,027	302,540	145,298
Gene/Genome (%)	85.77	87.94	85.71	87.66
Gene density (genes/Mbp)	959	940	963	946
Average gene length (bp)	895	936	890	927
GC content (%)	57.15	57.34	57.16	57.34
N50	2,126,219	1,185,635	2,117,144	1,177,787
L50	1	1	1	1
BUSCO completeness	99.80%		99.40%	
Sequencing platform	Oxford Nanopore		Shotgun Sequencing	
Coverage	591×		9×	

**Table 3:** Statistical data of the genome characteristics of *Brucella melitensis* strains IMHB1 and 16M.

Category	IMHB1		16M	
	Chromosome I	Chromosome II	Chromosome I	Chromosome II
CDS	2038	1114	2039	1114
tRNA	41	14	40	14
tmRNA	1	0	1	0
rRNA	6	3	6	3
ncRNA	27	12	27	12
oriC	2	1	2	1
CRISPR array	0	0	0	0
Repeat elements	80	38	70	33
Prophage	1	0	1	0

### 3.4 Genomic functional features of strain IMHB1

**Figure 5:**
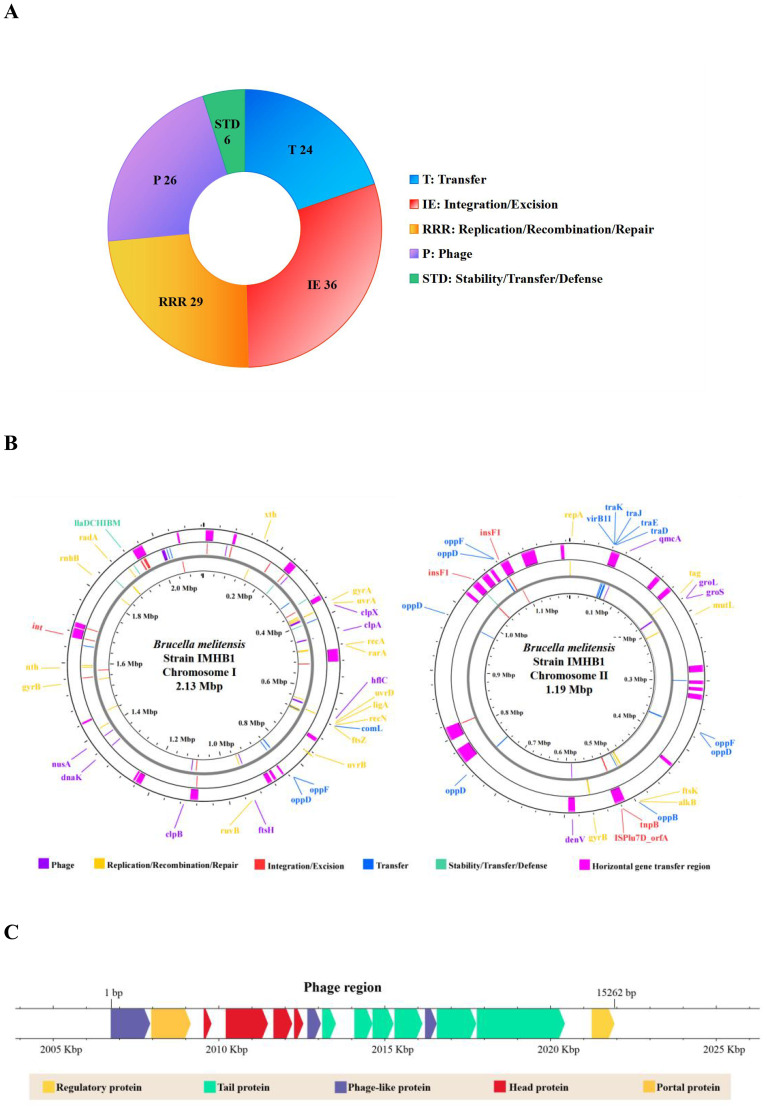
Genomic functional features of *Brucella melitensis* strain IMHB1. **(A)** Composition of 121 mobile genetic elements (MGEs). **(B)** Distribution of 121 MGEs and 36 putative horizontal gene transfer (HGT) regions across the two chromosomes of strain IMHB1. **(C)** Location and composition of the integrated phage genome on chromosome I of strain IMHB1.

The functional genomic features of strain IMHB1 encompassed mobile genetic elements (MGEs), putative horizontal gene transfer (HGT) regions, and prophage regions. The mobile orthologous groups database (MobileOG-db) is an interactive database that catalogs a wide range of proteins regulating the lifecycle of MGEs in bacteria. Five essential functional categories of MGEs were defined: (i) integration and excision (IE) from one genetic locus to another; (ii) replication, recombination, or nucleic acid repair (RRR); (iii) interorganism transfer (T); (iv) element stability, transfer, or defense (STD); and (v) phage-specific (P) biological processes. The MGEs analysis was performed using thresholds of >60% identity, an E-value of <1e-5, and evidence from manual, homology, and keyword searches. A total of 121 MGEs were identified in the genome of *B. melitensis* strain IMHB1 (**Figure 5A**), comprising 36 IEs, 29 RRRs, 24 transfers, 6 STDs, and 26 Ps (all MGE regions are shown in **Figure 5B**).

HGT events were detected using Alien Hunter in Proksee, revealing their distribution across both chromosomes. 17 putative HGT regions, with a combined size of 228,246 bp, were identified on chromosome I, and 19 regions spanning 199,613 bp were detected on chromosome II (**Figure 5B**; **Supplementary Table S3**). A prophage region spanning 2,006,080–2,022,067 bp was predicted by PHASTEST with a score of 150, indicating an intact prophage region (score > 90). This region contained 21 ORFs, of which 15 encoded four head proteins, six tail proteins, one regulatory protein, two phage-like proteins, one portal protein, and one terminase (**Figure 5C**).

### 3.5 Biological functional annotation of strain IMHB1

**Figure 6:**
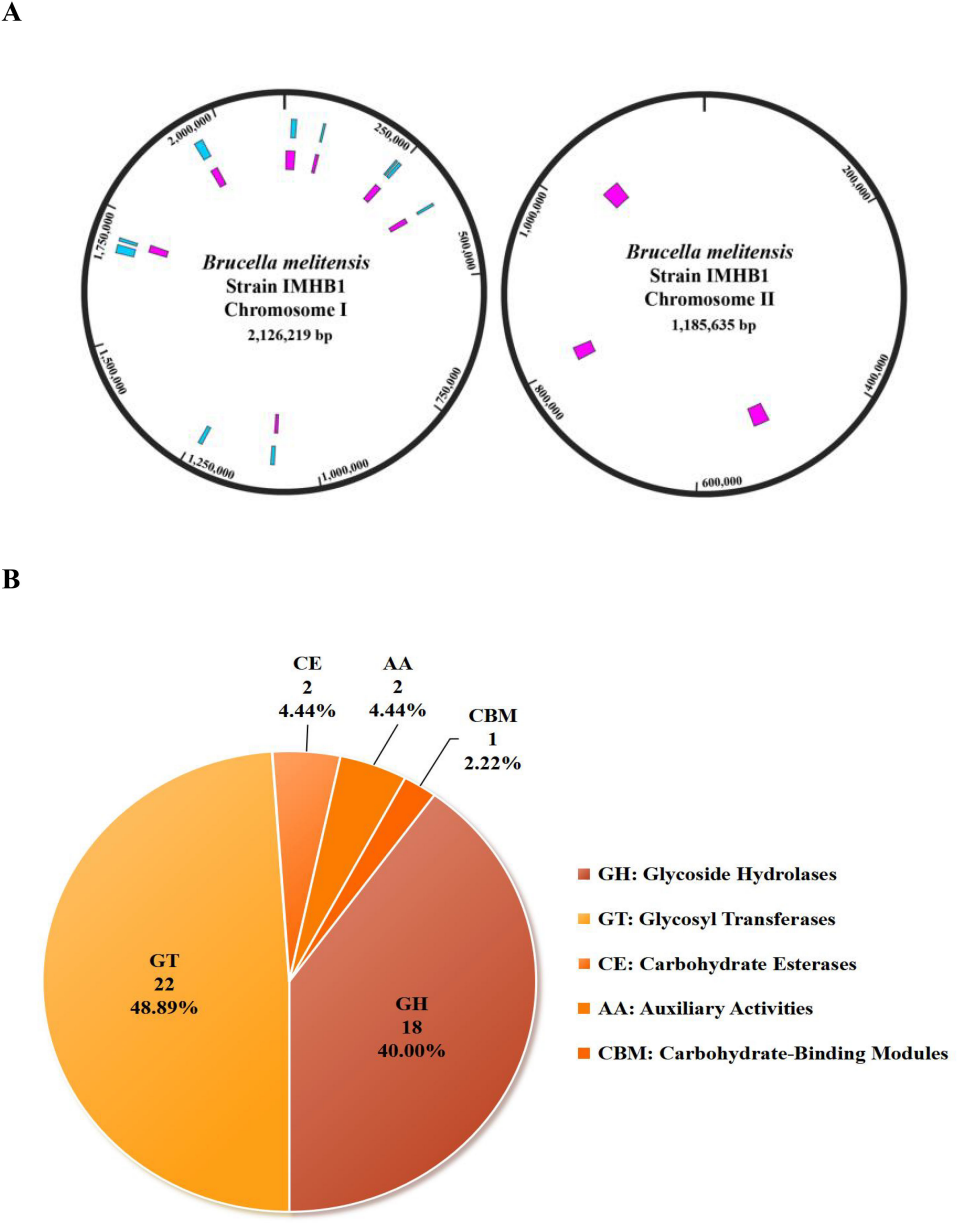
Biological functional characteristics of *Brucella melitensis* strain IMHB1. **(A)** Distribution of predicted gene islands across the two chromosomes of strain IMHB1. Pink represents gene islands predicted by the IslandPath-DIMOB method, and blue represents those predicted by the SIGI-HMM method. **(B)** Composition of 45 CAZymes predicted using the CAZy database.

Functional annotation of strain IMHB1 identified features related to gene islands, carbohydrate-active enzymes, biosynthetic gene clusters, and antibiotic resistance genes. Seven GIs were predicted to be located on chromosome I (comprising 113 genes) and three on chromosome II (containing 63 genes) by the IslandPath-DAMOB method (**Figure 6A**). To identify potential virulence islands, genes inside and outside the predicted GIs were annotated against the VFDB database, indicating that no significant enrichment of virulence determinants was observed outside the predicted GIs (*P* > 0.05).

A total of 68 and 123 CAZymes were predicted by HMMER and DIAMOND, respectively. The intersection of these two sets revealed 45 overlapping CAZymes, including 18 glycoside hydrolases, 22 glycosyl transferases, 2 auxiliary activities, 2 carbohydrate esterases, and 1 carbohydrate-binding module (**Figure 6B**; **Supplementary Table S4**). Compared with *B. melitensis* 16M, IMHB1 revealed a minor reduction in one glycoside hydrolase family. Three BGCs were detected on chromosome I, including arylpolyene (111,706–152,887 bp), terpene (160,013–180,846 bp), and β-lactone (1,416,579–1,444,388 bp), all of which shared 99% similarity with *B. melitensis* 16M.

The genome of the IMHB1 strain was screened for antibiotic resistance genes, revealing a high-confidence match to *mprF* with 99.66% identity, while three additional putative resistance genes (q*acG*, a*deF*, and *fosXCC*) were detected, each exhibiting <60% similarity to known resistance determinants (**Table 4**). An additional panel of 14 *Brucella* genomes identified by cgMLST genotyping was screened, showing that the prevalence of the *mprF* resistance gene was 100%.

**Table 4:** Predicted antibiotic resistance genes in *Brucella melitensis* IMHB1.

Location	Pass bitscore	Hit bitscore	Cut-off	Identity (%)	Hit ARO	Resistance mechanism	Antibiotic class	Model type	Prevalence (%) (*n* = 15)
Chromosome I	75	95.1	Strict	42.31	QacG	Antibiotic efflux	Benzalkonium chloride	Protein homolog	100.00
	750	874.8	Strict	46.82	AdeF	Antibiotic efflux	Tetracycline	Protein homolog	100.00
Chromosome II	750	793.1	Strict	43.57	AdeF	Antibiotic efflux	Tetracycline	Protein homolog	86.67
	150	160.2	Strict	55.56	FosXCC	Antibiotic inactivation	Fosfomycin	Protein homolog	100.00
	1650	1706.8	Strict	99.66	MprF	Antibiotic target alteration	Defensin	Protein homolog	100.00

**Figure 7:**
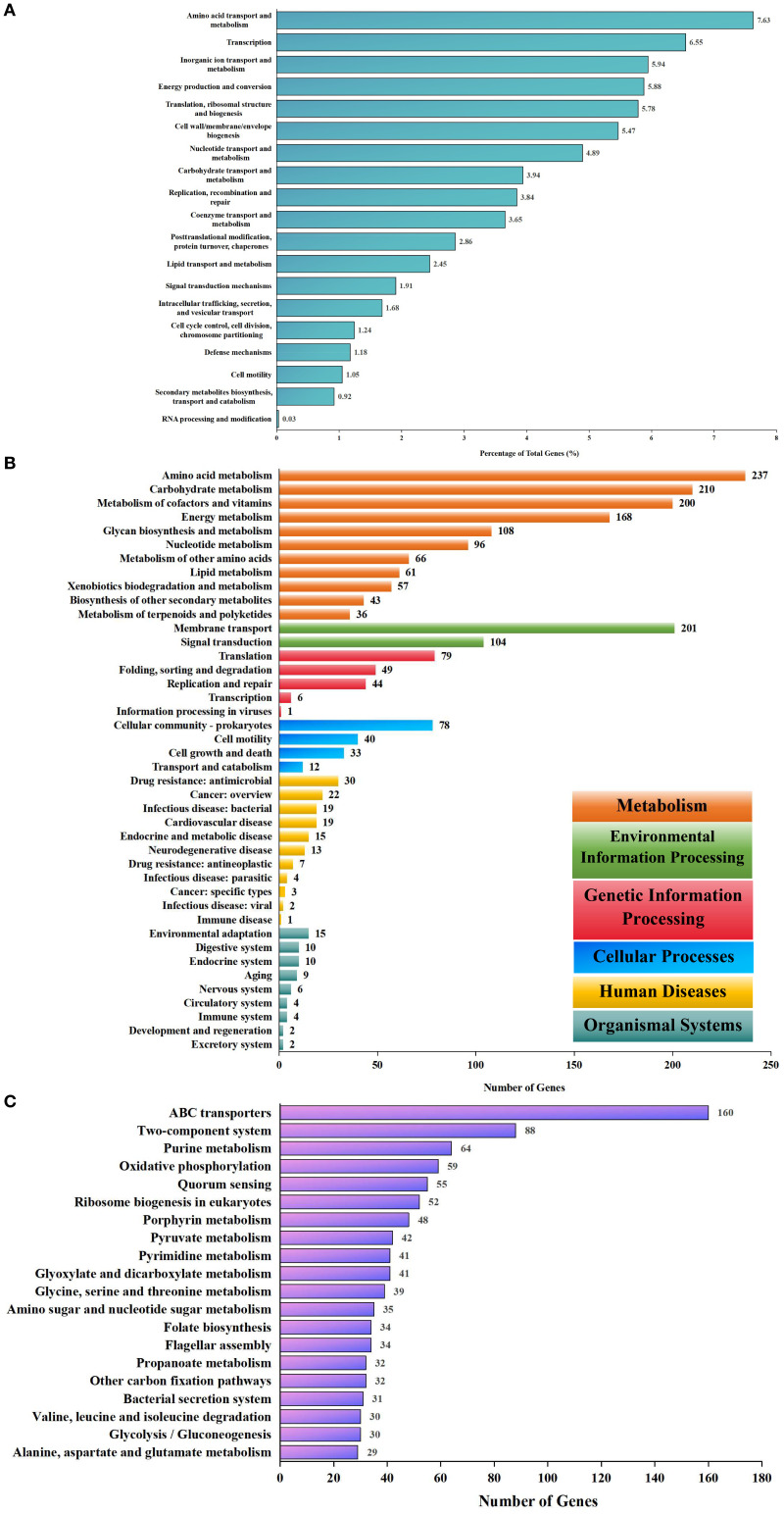
Biological functional annotations of *Brucella melitensis* strain IMHB1. **(A)** EggNOG database annotation of genes on the two chromosomes of strain IMHB1. The main functional categories included amino acid transport and metabolism; transcription; inorganic ion transport and metabolism; energy production and conversion; and translation, ribosomal structure, and biogenesis. **(B)** KEGG database annotation of genes on the two chromosomes at level two. The genes were classified into 11 metabolism classes, 11 human disease classes, nine organismal system classes, five genetic information processing classes, four cellular process classes, and two environmental information processing classes. **(C)** KEGG database annotation of genes on the two chromosomes at level three. The major pathways included ABC transporters, two-component systems, purine metabolism, oxidative phosphorylation, and quorum sensing.

Functional annotation using eggNOG and KEGG indicated broad metabolic capabilities. Annotation of the eggNOG identified 20 COGs; however, most annotated proteins (556) lacked functional characterization. The most abundant functional categories included amino acid transport and metabolism, transcription, inorganic ion transport and metabolism, energy production and conversion, translation, ribosomal structure, and biogenesis (**Figure 7A**). Furthermore, annotation by the KEGG database revealed 11 metabolism classes, 11 human disease classes, 9 organismal system classes, 5 genetic information processing classes, 4 cellular process classes, and 2 environmental information processing classes (**Figure 7B**). The top 20 of the 252 KEGG pathways included ABC transporters, two-component systems, purine metabolism, oxidative phosphorylation, and quorum sensing (**Figure 7C**). Compared to *B. melitensis* 16M, strain IMHB1 contained CbiM, ArgT, and HisJ in the ABC transporter pathway; PcaF in benzoate degradation; and LepB in the legionellosis pathway, while lacking BioM in the ABC transporter pathway, AdeB in the beta-lactam resistance pathway, and YxdM in the two-component system pathway.

### 3.6 Prediction of pathogenic molecules of strain IMHB1

Virulence factors of strain IMHB1 were predicted using BLASTP against the VFDB core dataset under a threshold of >80% identity, alignment length of >100 amino acids, and E-value < 1e-10. A total of 66 virulence factors were identified, including 6 factors related to adherence, 25 to effector delivery systems, 33 to immune modulation, and 2 to regulation, respectively (**Table 5**). Compared with *B. melitensis* 16M, strain IMHB1 lacked BtaF (VF1343) in the adherence category.

**Table 5:** Categories and functional characteristics of predicted virulence factors in *Brucella melitensis* IMHB1.

Gene ID	Identity (%)	Length	E-value	VF Gene ID	VF gene name	VF name and ID	VF catagory
IMHB1_2_56	100	172	4.8e-126	VFG041369	VirB12	VirB type IV secretion system (VF0365)	Effector delivery system (VFC0086)
IMHB1_2_57	100	361	0	VFG002218	VirB11		
IMHB1_2_58	99.211	380	0	VFG002217	VirB10		
IMHB1_2_59	99.298	285	0	VFG002216	VirB9		
IMHB1_2_60	100	239	0	VFG002215	VirB8		
IMHB1_2_62	99.424	347	0	VFG002213	VirB6		
IMHB1_2_63	100	238	0	VFG002212	VirB5		
IMHB1_2_64	99.88	831	0	VFG002211	VirB4		
IMHB1_2_65	100	116	2.23e-83	VFG002210	VirB3		
IMHB1_2_66	100	105	2.77e-72	VFG002209	VirB2		
IMHB1_2_67	100	238	4.09e-178	VFG002208	VirB1		
IMHB1_1_111	100	137	3.72e-96	VFG051167	BspC	T4SS secreted effectors (VF0695)	
IMHB1_1_288	99.874	1582	0	VFG051244	BPE043		
IMHB1_1_301	99.522	418	0	VFG041432	VceC		
IMHB1_1_503	100	242	7.69e-176	VFG051262	BPE275		
IMHB1_1_507	99.429	175	1e-129	VFG045340	RicA		
IMHB1_1_701	100	190	1.9e-142	VFG051271	SepA		
IMHB1_1_738	98.81	168	3.69e-120	VFG051202	BspL		
IMHB1_1_843	100	105	1.39e-75	VFG041431	VceA		
IMHB1_1_860	100	264	0	VFG051176	BspE		
IMHB1_1_1111	100	428	0	VFG051185	BspF		
IMHB1_1_1162	100	153	1.26e-107	VFG051235	BPE005		
IMHB1_1_1984	100	191	3.24e-137	VFG051149	BspA		
IMHB1_1_2013	100	187	4.28e-136	VFG051158	BspB		
IMHB1_2_122	98.693	153	2.77e-102	VFG051253	BPE123		
IMHB1_1_31	100	255	0	VFG011439	LpxE	LPS (VF0367)	Immune modulation (VFC0258)
IMHB1_1_132	99.675	308	0	VFG011419	HtrB		
IMHB1_1_251	99.512	410	0	VFG011444	WboA		
IMHB1_1_252	99.807	519	0	VFG011515	WbdA		
IMHB1_1_392	100	277	0	VFG011414	KdsA		
IMHB1_1_407	100	395	0	VFG011409	LpxB		
IMHB1_1_409	100	278	0	VFG011404	LpxA		
IMHB1_1_410	100	157	1.44e-115	VFG011399	FabZ		
IMHB1_1_727	99.718	354	0	VFG011510	LpsB/LpcC		
IMHB1_1_1376	100	251	0	VFG011424	KdsB		
IMHB1_1_1395	99.816	543	0	VFG002220	Pgm		
IMHB1_1_1847	100	334	0	VFG011519	WbpL		
IMHB1_1_1852	100	259	0	VFG002230	WbkC		
IMHB1_1_1853	100	284	0	VFG002229	WbkB		
IMHB1_1_1854	99.603	252	0	VFG002228	Wzt		
IMHB1_1_1855	100	260	0	VFG002227	Wzm		
IMHB1_1_1856	100	367	0	VFG002226	Per		
IMHB1_1_1857	100	362	0	VFG002225	Gmd		
IMHB1_1_1863	100	372	0	VFG002224	WbkA		
IMHB1_1_1869	99.778	451	0	VFG002223	Pmm		
IMHB1_1_1870	99.779	453	0	VFG002222	ManCoAg		
IMHB1_1_1871	100	390	0	VFG002221	ManAoAg		
IMHB1_1_1872	100	369	0	VFG011453	WbpZ		
IMHB1_1_1948	99.858	703	0	VFG011505	LpsA		
IMHB1_2_204	99.776	446	0	VFG011535	WaaA/KdtA		
IMHB1_2_205	99.707	341	0	VFG011533	LpxK		
IMHB1_2_324	100	471	0	VFG011528	ManCcore		
IMHB1_1_411	100	351	0	VFG011394	LpxD		
IMHB1_1_656	100	286	0	VFG011389	LpxC		
IMHB1_2_325	100	477	0	VFG011524	ManBcore		
IMHB1_1_1445	99.651	2867	0	VFG002219	Cgs	CβG (VF0366)	
IMHB1_1_1608	100	275	0	VFG045465	BtpA	BtpA/Btp1/TcpB (VF0412)	
IMHB1_1_26	99.315	292	0	VFG045466	BtpB	BtpB (VF0522)	
IMHB1_2_166	99.235	523	0	VFG051123	BmaA	BmaA (VF1339)	Adherence (VFC0001)
IMHB1_1_1172	99.823	565	0	VFG051125	BmaB/OmaA	BmaB/OmaA (VF1340)	
IMHB1_2_1065	99.843	635	0	VFG051131	BmaC	BmaC (VF1341)	
IMHB1_1_1409	99.148	352	0	VFG051134	BtaE	BtaE (VF1342)	
IMHB1_1_1166	96.748	123	1.61e-76	VFG051113	BigA	BigA (VF1344)	
IMHB1_1_1169	99.807	518	0	VFG051120	BigB	BigB (VF1345)	
IMHB1_1_1251	100	239	2.95e-178	VFG011626	BvrR	BvrR-BvrS (VF0368)	Regulation (VFC0301)
IMHB1_1_1252	100	601	0	VFG011631	BvrS		

The interactions between the pathogen and host were analyzed against the PHI database, indicating that 18 proteins have experimentally demonstrated effects on the pathogenicity of *Brucella*, four proteins have no reported impact (**Table 6**), and four proteins have roles in virulence that remain controversial. All experimental evidence was derived from studies employing *Mus musculus* as the host.

**Table 6:** Predicted interaction between *Brucella melitensis* strain IMHB1 and host.

Gene name	PHI-base entry	E-value	Identity (%)	Coverage (%)	Mutant phenotype	Pathogen species	Host species
BlxR	PHI:7609	3.10e-134	99.57	100.00	Unaffected pathogenicity	*Brucella abortus*	*Mus musculus*
BMEI1329	PHI:5423	4.20e-131	100.00	100.00	Reduced virulence	*Brucella melitensis*	*Mus musculus*
BpdA	PHI:3645	0	99.90	100.00	Loss of pathogenicity	*Brucella melitensis*	*Mus musculus*
	PHI:7184				Reduced virulence		
BpdB	PHI:3646	9.50e-304	100.00	96.69	Reduced virulence	*Brucella melitensis*	*Mus musculus*
BtaE	PHI:3891	1.10e-206	95.89	91.96	Reduced virulence	*Brucella suis*	*Mus musculus*
BtpB	PHI:3722	1.30e-161	98.92	94.86	Effector	*Brucella abortus*	*Mus musculus*
CgsB	PHI:3647	5.10e-203	100.00	93.18	Increased virulence	*Brucella melitensis*	*Mus musculus*
	PHI:7185				Reduced virulence		
InvA	PHI:4915	1.90E-107	100.00	100.00	Reduced virulence	*Brucella melitensis*	*Mus musculus*
Lon	PHI:8922	0	99.51	100.00	Unaffected pathogenicity	*Brucella abortus*	*Mus musculus*
	PHI:8923				Reduced virulence		
Pyk	PHI:6684	2.10e-267	99.58	100.00	Reduced virulence	*Brucella abortus*	*Mus musculus*
RegM	PHI:4524	2.90e-254	99.78	88.97	Unaffected pathogenicity	*Brucella melitensis*	*Mus musculus*
RfbE	PHI:7603	1.00e-135	99.21	100.00	Unaffected pathogenicity	*Brucella abortus*	*Mus musculus*
	PHI:7604				Reduced virulence		
SepA	PHI:3829	7.60e-107	99.47	100.00	Unaffected pathogenicity	*Brucella abortus*	*Mus musculus*
					Reduced virulence		
TcpB	PHI:6367	4.10e-96	98.18	100.00	Reduced virulence	*Brucella melitensis*	*Mus musculus*
VtlR	PHI:5012	2.50e-173	100.00	100.00	Reduced virulence	*Brucella abortus*	*Mus musculus*
WadB	PHI:3070	3.70e-135	100.00	100.00	Reduced virulence	*Brucella abortus*	*Mus musculus*
Bab2_0612	PHI:7201	1.50e-39	80.22	97.85	Reduced virulence	*Brucella abortus*	*Mus musculus*
Bab2_0879	PHI:7202	4.80e-208	99.71	100.00	Unaffected pathogenicity	*Brucella abortus*	*Mus musculus*
BveA	PHI:6489	4.50e-278	100.00	100.00	Reduced virulence	*Brucella melitensis*	*Mus musculus*
IbpA	PHI:4102	1.50e-84	99.36	100.00	Unaffected pathogenicity	*Brucella suis*	*Mus musculus*
LovhK	PHI:3306	4.70e-286	100.00	100.00	Loss of pathogenicity	*Brucella abortus*	*Mus musculus*
TceSR	PHI:4834	7.30e-268	100.00	100.00	Reduced virulence	*Brucella melitensis*	*Mus musculus*
VirB1	PHI:7604	2.90e-132	99.58	100.00	Reduced virulence	*Brucella abortus*	*Mus musculus*
VirB2	PHI:7605	4.30e-51	100.00	100.00	Reduced virulence	*Brucella abortus*	*Mus musculus*
VirB3	PHI:7606	1.60e-59	100.00	100.00	Reduced virulence	*Brucella abortus*	*Mus musculus*
VjbR	PHI:7607	1.20e-150	100.00	100.00	Reduced virulence	*Brucella abortus*	*Mus musculus*

## 4 Discussion

Although brucellosis is a recognized zoonotic disease, the functional genomic characteristics of *Brucella* strains in specific regions remains poorly characterized, hindering the development of targeted public health control measures. In 2002, the genome of *B. melitensis* was sequenced for the first time using the shotgun approach (DelVecchio et al., 2002). However, few reports exist on the genomic characteristics of *Brucella* in Hulunbuir, Inner Mongolia, China. We previously reported the molecular epidemiological characteristics of 20 *B. melitensis* strains associated with arthritis in Hulunbuir based on Illumina NovaSeq sequencing data (Zhu et al., 2024). The complete genomic structure and functional characteristics of *B. melitensis* from Hulunbuir, based on Oxford Nanopore sequencing technology, were reported for the first time.

In this study, a *B. melitensis* strain IMHB1 was isolated from the blood of a patient with brucellosis. Genome sequencing revealing two chromosomes, totaling 3.32 Mbp, with no plasmid detected. The GC content of this genome was 57.22%, and 3152 coding genes were predicted, covering 86.55% of the genome. Of these, 94.26% were functionally annotated in the eggNOG database, showing high consistency with *B. melitensis* 16M. Previous studies have demonstrated that the highly conserved genome of *Brucella* represents a stable adaptive strategy evolved through long-term association with its natural hosts (Salmon-Divon et al., 2018a; Salmon-Divon et al., 2018b). The absence of significant mutations in the clinical isolates suggests that large-scale genomic recombination is not a prerequisite for cross-species transmission. Correspondingly, the prediction of highly conserved virulence factors indicates that *Brucella* employs similar mechanisms for intracellular survival, immune evasion, and pathogenesis in diverse hosts (Moreno, 2014; Celli, 2019). This genomic stability may contribute to the persistent challenge of brucellosis as a zoonotic disease.

The PHASTEST database predicted an intact prophage genome (15.2 kb) in *B. melitensis* IMHB1, whereas a shorter prophage region (11.7 kb) was identified in *B. melitensis* 16M. This variation provides evolutionary evidence for historical genetic recombination between bacteriophages and *Brucella*. *PhoP* in the prophage genome and *phoQ* in the *Brucella genome* are tandemly arranged and constitute a two-component system (TCS). PhoQ senses Mg^2+^, cationic antimicrobial peptides, and short-chain fatty acids via autophosphorylation, activating the transcription factor PhoP. Phosphorylated PhoP regulates gene expression to enhance bacterial resistance to antimicrobial agents and nutrient stress (Montagne et al., 2001; Groisman et al., 2021).

Currently, research on the PhoQ/PhoP TSC has predominantly focused on *Enterobacteriaceae*, with few reports in *Brucella*. The prediction that a prophage-integrated PhoQ/PhoP TCS enhances bacterial resistance remains computationally unconfirmed. Therefore, future studies employing RNA-seq and gene knockout techniques are essential to validate the co-expression of this system and elucidate its impact on *Brucella* virulence.

Under strict RGI matching, the resistance gene *mprF* was identified with high confidence. MprF is a large membrane protein that modifies the anionic phospholipid phosphatidylglycerol on the membrane surface, thereby reducing the affinity for cationic antimicrobial peptides, such as defensin, and conferring resistance to innate host defenses and cationic antibiotics (Ernst et al., 2009). An antibody has been reported to block the physiological function of MprF, rendering MRSA sensitive to cationic antimicrobial peptides and antibiotics and reducing MRSA survival in human phagocytes (Slavetinsky et al., 2022). The functional blockade of *mprF* may be a novel strategy for the clinical treatment of brucellosis.

The three additional putative resistance genes identified in the IMHB1 strain exhibited <60% sequence identity with their reference sequences in the CARD database. This level of homology is generally considered insufficient to confer a definitive resistance phenotype. Nonetheless, the detection of these homologs implies the presence of evolutionarily related sequences within the genome, which may represent divergent resistance genes, pseudogenes, or ancestral genetic elements that have not acquired full resistance capability (Alcock et al., 2023). As these predictions are based solely on protein homology, future studies employing functional validation, such as gene expression profiling under antibiotic stress or cloning coupled with heterologous expression, are essential to elucidate their potential role in antimicrobial resistance.

Although our study provides a comprehensive genomic characterization of *B. melitensis* IMHB1 to elucidate its potential functions, some limitations should be acknowledged. The primary limitation is its *in silico* and predictive nature. All genomic features and functional analyses were based on DNA or protein sequence homology. Therefore, these predictions require subsequent *in vitro* and *in vivo* experimental validation to confirm their functional expression and biological significance during actual infection. On the other hand, the findings from a single clinical isolate, although insightful, are limited in their broader applicability. Specific genomic variations, such as those distinguishing it from the *B. melitensis* 16M strain, may represent singular occurrences rather than patterns common across *Brucella* spp.

Despite these limitations, the genomic insights garnered from this study establish a critical foundation for several pivotal research avenues. The genomic conservation observed in *Brucella* suggests that the core mechanisms underlying intracellular survival, replication, immune evasion, and pathogenesis may be shared across species and hosts. To fully decipher this pathogenic blueprint, future studies should prioritize large-scale pan-genome and comparative genomic analyses. Such efforts will be instrumental in refining the phylogeny of *Brucella*, identifying genuine genetic markers of tissue tropism and disease severity, and distinguishing the stable core genomes from the dynamic accessory genomes. Furthermore, to bridge the gap between genomic potential and functional reality, it is essential to integrate transcriptomic and proteomic data from bacteria cultured under conditions that mimic intracellular niches. This multi-omics approach will unequivocally reveal the pathways actively employed during infection. Addressing these gaps will move beyond genomic prediction toward a mechanistic understanding of brucellosis pathogenesis and facilitate the development of novel diagnostic and therapeutic strategies.

## 5 Conclusion

In summary, this study presents a complete genome map of *B. melitensis* biotype III strain IMHB1, closely related to cgST-588, a clinical isolate from China. The multi-faceted genomic characterization delineated its architecture, comprising two circular chromosomes (2.13 Mbp and 1.19 Mbp), and identified key genetic elements, including 121 MGEs, 36 putative HGT regions, and a prophage region. Functional annotation further revealed 10 genomic islands, 45 CAZymes, three BGCs, and four antibiotic resistance genes, along with annotation in 20 eggNOG categories and 252 KEGG pathways. Notably, 66 virulence factors were identified, including 18 proteins with experimentally verified roles in pathogen-host interactions. Collectively, these findings provide a valuable genomic resource and multilayered insights into *B. melitensis*, which will support future studies aimed at improving the diagnosis and treatment of brucellosis.

## Data Availability

The datasets presented in this study can be found in online repositories. The names of the repository/repositories and accession number(s) can be found below: https://www.ncbi.nlm.nih.gov/, CP195256-CP195257.
