# Supplementary figures and images for: Complete genome assembly and functional characterization of Brucella melitensis strain IMHB1 from a clinical isolate in Inner Mongolia, China
Source: Front Cell Infect Microbiol. 2025 Dec 9;15:1653521. doi: 10.3389/fcimb.2025.1653521 (PMC12722894; doi:10.3389/fcimb.2025.1653521)

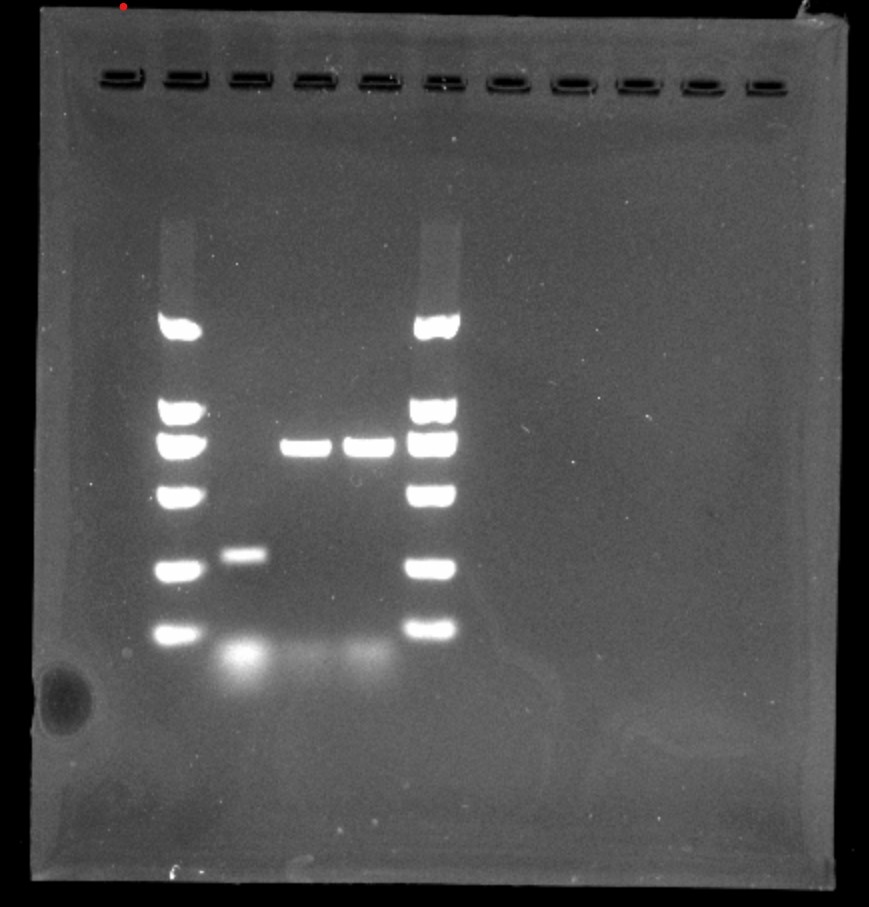

Supplement: Supplementary file 1 [file Image1.jpeg]
