# Supplementary material for: Complete genome assembly and functional characterization of Brucella melitensis strain IMHB1 from a clinical isolate in Inner Mongolia, China
Source: Front Cell Infect Microbiol. 2025 Dec 9;15:1653521. doi: 10.3389/fcimb.2025.1653521 (PMC12722894; doi:10.3389/fcimb.2025.1653521)
Supplement: Supplementary file 2 [file Table1.docx]

| GenBank assembly ID | Rename | Geographical location | Host | Species |
| --- | --- | --- | --- | --- |
| GCF_003516045.1 | IM01 | Inner Mongolia, China | *Homo sapiens* | *Brucella melitensis* |
| GCF_003516065.1 | IM02 | Inner Mongolia, China | *Homo sapiens* | *Brucella melitensis* |
| GCF_003516085.1 | IM03 | Inner Mongolia, China | *Homo sapiens* | *Brucella melitensis* |
| GCF_004208655.1 | IM04 | Inner Mongolia, China | *Homo sapiens* | *Brucella melitensis* |
| GCF_004208675.1 | IM05 | Inner Mongolia, China | *Homo sapiens* | *Brucella melitensis* |
| GCF_004208695.1 | IM06 | Inner Mongolia, China | *Homo sapiens* | *Brucella melitensis* |
| GCF_003856415.1 | IM07 | Inner Mongolia, China | *Homo sapiens* | *Brucella melitensis* |
| GCF_038420175.1 | IM08 | Inner Mongolia, China | *Capra hircus* | *Brucella melitensis* |
| GCF_023796775.1 | IM09 | Inner Mongolia, China | *Homo sapiens* | *Brucella melitensis* |
| GCF_002763615.1 | QH01 | Qinghai, China | *Bos grunniens* | *Brucella melitensis* |
| GCF_036320855.1 | HB01 | Hebei, China | *Homo sapiens* | *Brucella melitensis* |
| GCF_027625455.1 | TB01 | Tibet, China | *Ovis aries* | *Brucella melitensis* |
| GCF_000007125.1 | *B. melitensis* 16M | Reference strains | *Ovis aries* | *Brucella melitensis* |
| GCF_000369945.1 | *B. abortus* 554 | Reference strains | *Bos taurus* | *Brucella abortus* |

Table S1 Summary of Brucella Genomes from the NCBI Database

| Process | Category | IMHB1 |
| --- | --- | --- |
| Nanopore sequencing data | Bases (bp) | 1,958,013,713 |
|  | Read number | 530,888 |
|  | Reads mean length (bp) | 3,688 |
|  | Reads N50 (bp) | 9,838 |
|  | Longest length (bp) | 155,286 |
| Illumina HiSeq sequencing data | Total reads (bp) | 10,099,588 |
|  | Clean reads (bp) | 10,099,588 |
|  | Percentage | 1 |
|  | %>Q20 | 99.46 |
|  | %>Q30 | 98.31 |
| Assembly data | Counts of scaffold sequences | 2 |
|  | Length of scaffold sequences (bp) | 2,126,219 |
|  |  | 1,185,635 |
|  | Sequencing Depth | 699 |
|  |  | 1,253 |

Table S2 Statistical data of sequencing and assembly

Table S3 Coordinates of horizontal gene transfer region

| **Location** | **Start** | **Stop** | **Size (bp)** | **Score** | **Threhold** |
| --- | --- | --- | --- | --- | --- |
| Chr Ⅰ | 3,415 | 22,506 | 19,091 | 0.44 | 18.60 |
|  | 77,848 | 82,970 | 5,122 | 0.32 | 18.60 |
|  | 234,822 | 252,076 | 17,254 | 0.36 | 18.60 |
|  | 348,873 | 357,484 | 8,611 | 0.38 | 18.60 |
|  | 488,061 | 521,817 | 33,756 | 0.24 | 18.60 |
|  | 723,976 | 733,990 | 10,014 | 0.25 | 18.60 |
|  | 844,688 | 852,746 | 8,058 | 0.21 | 18.60 |
|  | 869,622 | 877,590 | 7,968 | 0.22 | 18.60 |
|  | 881,071 | 893,123 | 12,052 | 0.50 | 18.60 |
|  | 1,078,354 | 1,096,400 | 18,046 | 0.45 | 18.60 |
|  | 1,230,445 | 1,240,719 | 10,274 | 0.67 | 18.60 |
|  | 1,241,803 | 1,248,535 | 6,732 | 0.24 | 18.60 |
|  | 1,437,726 | 1,444,611 | 6,885 | 0.19 | 18.60 |
|  | 1,665,301 | 1,688,421 | 23,120 | 0.35 | 18.60 |
|  | 1,694,432 | 1,703,372 | 8,940 | 0.43 | 18.60 |
|  | 1,937,287 | 1,963,858 | 26,571 | 0.40 | 18.60 |
|  | 2,056,703 | 2,062,455 | 5,752 | 0.32 | 18.60 |
| Chr Ⅱ | 59,288 | 70,079 | 10,791 | 0.26 | 16.29 |
|  | 131,575 | 138,876 | 7,301 | 0.18 | 16.29 |
|  | 151,232 | 161,811 | 10,579 | 0.44 | 16.29 |
|  | 277,465 | 288,203 | 10,738 | 0.20 | 16.29 |
|  | 296,745 | 304,892 | 8,147 | 0.19 | 16.29 |
|  | 309,381 | 314,356 | 4,975 | 0.17 | 16.29 |
|  | 318,274 | 328,651 | 10,377 | 0.20 | 16.29 |
|  | 426,423 | 432,151 | 5,728 | 0.23 | 16.29 |
|  | 512,371 | 525,308 | 12,937 | 0.35 | 16.29 |
|  | 582,719 | 593,120 | 10,401 | 0.28 | 16.29 |
|  | 761,662 | 781,093 | 19,431 | 0.18 | 16.29 |
|  | 798,029 | 817,999 | 19,970 | 0.31 | 16.29 |
|  | 1,018,009 | 1,022,963 | 4,954 | 0.27 | 16.29 |
|  | 1,030,700 | 1,039,946 | 9,246 | 0.25 | 16.29 |
|  | 1,049,726 | 1,059,560 | 9,834 | 0.20 | 16.29 |
|  | 1,064,588 | 1,071,693 | 7,105 | 0.20 | 16.29 |
|  | 1,083,098 | 1,092,371 | 9,273 | 0.62 | 16.29 |
|  | 1,116,891 | 1,135,253 | 18,362 | 0.36 | 16.29 |
|  | 1,170,488 | 1,179,952 | 9,464 | 0.21 | 16.29 |

Table S4 CAZymes family prediction

| Gene ID | CAZymes Family |
| --- | --- |
| Strain IMHB1_1_844 | AA3_2 |
| Strain IMHB1_1_1201 | AA3_2 |
| Strain IMHB1_1_167 | CBM50 |
| Strain IMHB1_1_656 | CE11 |
| Strain IMHB1_2_834 | CE9 |
| Strain IMHB1_2_67 | GH23 |
| Strain IMHB1_1_1235 | GH102 |
| Strain IMHB1_1_1009 | GH103 |
| Strain IMHB1_1_1369 | GH103 |
| Strain IMHB1_1_678 | GH103 |
| Strain IMHB1_2_68 | GH103 |
| Strain IMHB1_1_137 | GH108 |
| Strain IMHB1_1_254 | GH108 |
| Strain IMHB1_2_393 | GH140 |
| Strain IMHB1_1_1445 | GH189 |
| Strain IMHB1_1_1972 | GH23 |
| Strain IMHB1_1_673 | GH23 |
| Strain IMHB1_1_736 | GH23 |
| Strain IMHB1_1_677 | GH25 |
| Strain IMHB1_2_443 | GH25 |
| Strain IMHB1_2_498 | GH26 |
| Strain IMHB1_1_158 | GH3 |
| Strain IMHB1_1_1587 | GH73 |
| Strain IMHB1_1_664 | GT119 |
| Strain IMHB1_1_1948 | GT25 |
| Strain IMHB1_1_407 | GT19 |
| Strain IMHB1_1_1653 | GT25 |
| Strain IMHB1_2_131 | GT2 |
| Strain IMHB1_2_388 | GT2 |
| Strain IMHB1_1_1676 | GT25 |
| Strain IMHB1_1_815 | GT25 |
| Strain IMHB1_2_100 | GT26 |
| Strain IMHB1_1_663 | GT28 |
| Strain IMHB1_2_204 | GT30 |
| Strain IMHB1_1_1863 | GT4 |
| Strain IMHB1_1_1872 | GT4 |
| Strain IMHB1_1_252 | GT4 |
| Strain IMHB1_1_727 | GT4 |
| Strain IMHB1_2_379 | GT4 |
| Strain IMHB1_2_380 | GT4 |
| Strain IMHB1_1_1451 | GT51 |
| Strain IMHB1_1_191 | GT51 |
| Strain IMHB1_1_1915 | GT51 |
| Strain IMHB1_1_963 | GT51 |
| Strain IMHB1_2_133 | GT83 |
